# Assessing the allocation of attention during visual search using digit-tracking, a calibration-free alternative to eye tracking

**DOI:** 10.1038/s41598-023-29133-7

**Published:** 2023-02-09

**Authors:** Yidong Yang, Lei Mo, Guillaume Lio, Yulong Huang, Thomas Perret, Angela Sirigu, Jean-René Duhamel

**Affiliations:** 1 Key Laboratory of Brain, Cognition and Education, Ministry of Education, South China Normal University, Guangzhou, 510631 China; 2 Institute of Cognitive Sciences Marc Jeannerod CNRS, UMR 5229, 69675 Bron, France; 3 IMind Center of Excellence for Autism, Le Vinatier Hospital, Bron, France

**Keywords:** Psychology, Visual system

## Abstract

Digit-tracking, a simple, calibration-free technique, has proven to be a good alternative to eye tracking in vision science. Participants view stimuli superimposed by Gaussian blur on a touchscreen interface and slide a finger across the display to locally sharpen an area the size of the foveal region just at the finger's position. Finger movements are recorded as an indicator of eye movements and attentional focus. Because of its simplicity and portability, this system has many potential applications in basic and applied research. Here we used digit-tracking to investigate visual search and replicated several known effects observed using different types of search arrays. Exploration patterns measured with digit-tracking during visual search of natural scenes were comparable to those previously reported for eye-tracking and constrained by similar saliency. Therefore, our results provide further evidence for the validity and relevance of digit-tracking for basic and applied research on vision and attention.

## Introduction

The role of attention mechanisms in human visual perception has long been a field of great interest in psychology and neuroscience. In attention research, eye movement recordings are often conducted in order to infer how attention is allocated to the different sources of information in the visual field^1–3^. Recently a new approach, named digit-tracking, was proposed to measure eye movements of an observer. Unlike traditional optical eye-tracking technology which directly measures eye position, this novel method records finger’s movements as a proxy for eye movements^4^. Specifically, a Gaussian-blurred image is presented on a touch-sensitive interface and can be locally unblurred by sliding a finger over it. The Gaussian blur is used to simulate peripheral vision of human retina and touching the screen models the high acuity of fovea. Observers have to touch the screen continuously to explore the stimuli, thus eye movements can be inferred from finger movements.

**Figure 1:**
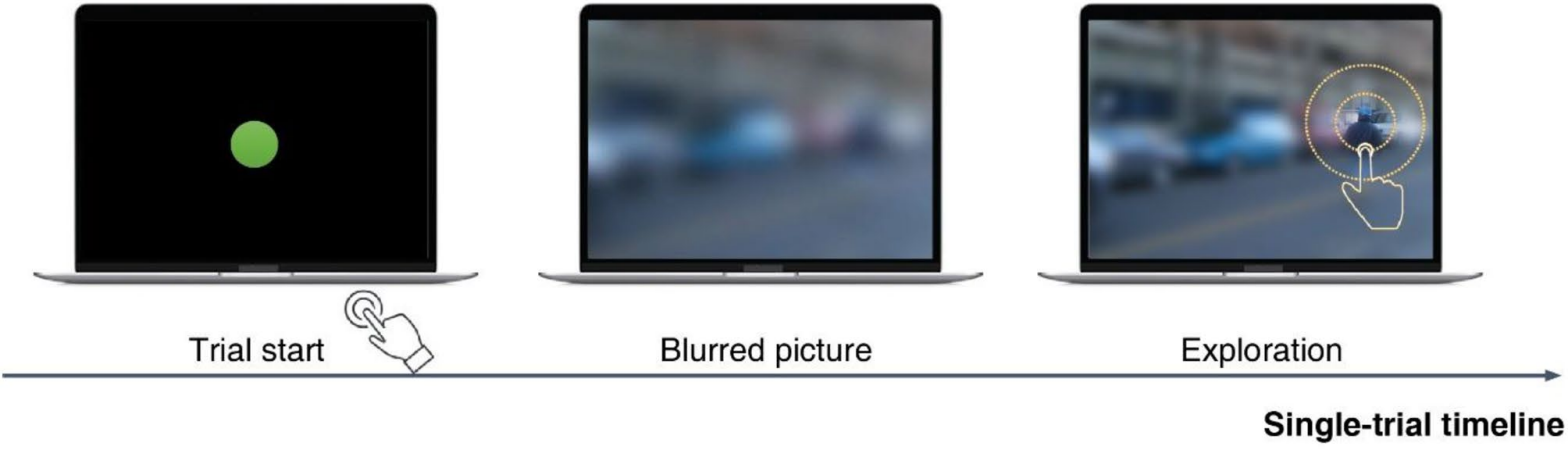
An illustration of the task. Participants saw a green button on the screen center. After touching the button, they could see the image superimposed by the gaussian blur. Touching the screen can sharpen an area around the finger, simulating the central vision. To clearly illustrate the Gaussian blur, the size of clear area here was larger than the size participants observed during the task.

This approach was validated by comparing it to standard eye-tracking on a free-viewing task. Attention maps derived from two methods were significantly correlated. Furthermore, the features driving attention during visual exploration, decomposed by a convolutional neural network, were similar in two methods^4^. Because digit-tracking is calibration-free, portable and easy to use, it could prove useful in a diverse range of experimental paradigms in visual science. Here, we assessed the robustness of digit-tracking for studies on visual search. A considerable amount of literature has been published on the factors that guide the deployment of attention during visual search and how people find targets^5–10^. Classic visual search studies use arrays of simple artificial items (abstract shapes, letters, etc.) to identify which low-level features are attention-guiding attributes^11–13^. Despite many robust and remarkable studies using artificial items, these stimuli lack visual complexity. Other studies using real-world objects or natural scenes have emerged in order to address the role of high-level features and scene context^14–19^. Whether observers will be guided by the same factors and will produce similar search patterns when tested with the digit-tracking interface remains to be established. Therefore, here we tested digit tracking on a range of visual search tasks (see an illustration of the task in Fig. 1).

In Experiment 1, we tested participants with several classic visual search tasks using artificial stimuli including two feature search arrays, one inefficient search array, one search array with heterogeneous distractors and a priming search task. In Experiment 2, we used arrays of real-world objects as stimuli to investigate the effect of the semantic relatedness between target and distractors. In Experiment 3, participants were asked to search for targets in natural scenes (pedestrians in an urban landscape) and tested the effect of blurring parameters on search performance, the correlation between digit and eye exploration patterns and the prediction performance of different computational models of attention. Results overall show that visual search with the digit-tracking method is in line with previously reported findings on search efficiency using arrays of artificial stimuli, the effect of semantic relatedness between target and distractors using arrays of real-world objects, and that searching for pedestrians embedded in natural scenes generates exploration patterns consistent with observations from standard eye-tracking studies and with models of search guidance that integrate scene context information.

## Results

### General consideration on digital exploration during visual search

**Figure 2:**
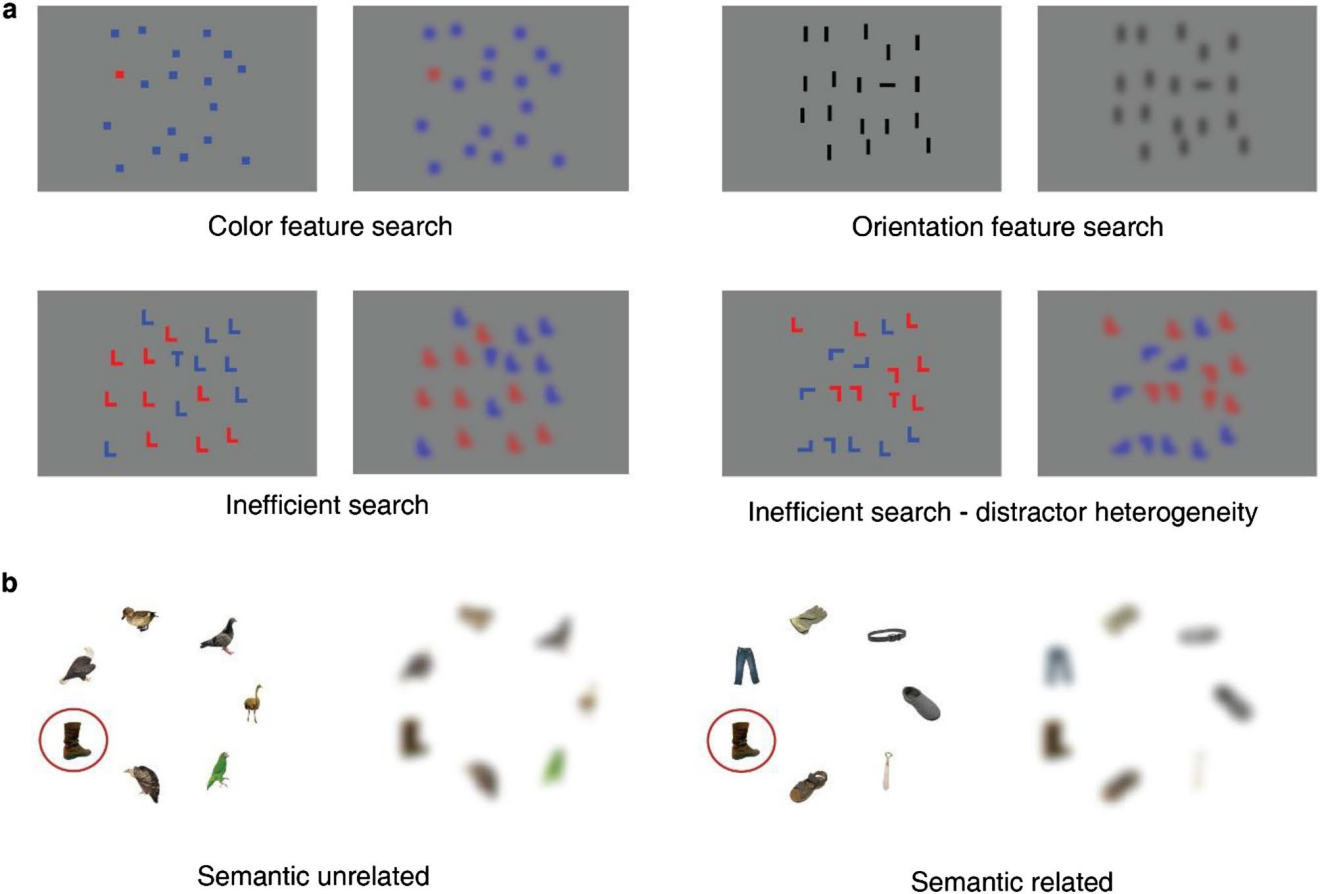
Examples of different search arrays before and after application of the blur filter. (**a**) Typical search arrays used in Experiment 1, including color feature search (1a, a red square as the target), orientation feature search (1b, a horizontal bar as the target), inefficient search with homogeneous (1c, a letter T as the target) and heterogeneous (1d, a letter T as the target) distractors. (**b**) Typical search arrays used in Experiment 2. The target was the boot, marked by a red rectangle here. Other objects were clothes in semantic related condition and birds in semantic unrelated condition.

**Figure 3:**
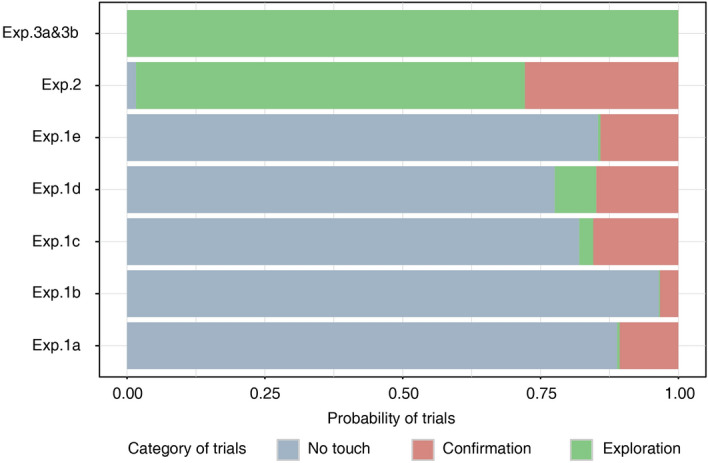
The bar plot of proportion of no touch, confirmation and exploration trials in different experiments. Participants barely touched the screen when searching in arbitrary arrays of items. In contrast, search in natural scenes generated more exploration.

Some examples of search stimuli used in Experiments 1 and 2 are shown in Fig. 2, in their native resolution and after blurring. The blurred image was created by applying a Gaussian filter of pre-defined standard deviation (see “Methods”) superimposed on the original image to simulate the peripheral vision. In Experiments 1 and 2, the task procedures required participants to report target presence or absence by pressing the corresponding response key. To search for the target, they were allowed, but not obligated, to explore the blurred display with their finger. In Experiment 3, we only presented target-present images and the participants were asked to localize the target. Search ended when the stimulus presentation software detected that the target remained unblurred for at least 700 ms (Experiment 3a) or by pressing the space key to confirm target detection (Experiment 3b). Thus, if target presence could be ascertained with the resolution of peripheral vision, participants could end the current trial by directly pressing the response key. Alternatively, participants could touch the screen to unblur a single item and confirm if it was the target. Therefore, visual search trials were classified into three groups: no-touch, confirmation and exploration trials. We defined a trial as a no-touch trial if the participant didn’t touch the screen at all. To distinguish confirmation and exploration trials, we labeled the target using a rectangle around it (i.e., target bounding box). Then we defined a trial as a confirmation trial if the tracking distance was shorter than the perimeter of the target bounding box or when more than 90 percent of tracking points fell into the target bounding box, otherwise, we defined it as an exploration trial. The resulting proportions of no-touch, confirmation, and exploration trials seemed related to task difficulty. Figure 3 shows the mean proportion of no touch, confirmation and exploration trials for the different search conditions. During feature searches (Experiments 1a and 1b), participants barely touched the screen, which indicates that these features could be extracted even when degraded by gaussian blur. High proportions of exploration trials are observed only when searching object arrays (Experiment 2) or natural scenes (Experiment 3, see example items in Fig. 5a), which means that the participants needed high resolution information to locate the target in stimuli of higher visual complexity.

### Experiment 1: Feature and inefficient search

The results of Experiments 1a to 1d were analyzed using two-way repeated measure ANOVA with target presence and either set size (1a to 1c) or distractor heterogeneity (1d) as factors. Response times replicated known effects using unblurred images. Color feature search was extremely easy as color information remain present in the blurred image (we did not attempt to mimic the rods to cones ratio from central to peripheral retina) and participants could easily distinguish between the red and blue items without ever needing to unblur the stimuli (Experiment 1a). The main effect of set size (6, 12 or 18 items) on response time was not significant, *F*(1.594, 49.419) = 0.811, *p* = 0.425, ${\eta }_{p}^{2}$ = 0.025, indicating that the search was efficient. In the orientation feature condition (Experiment 1b), response time increased with set size, *F*(2, 62) = 16.528, *p* < 0.001, ${\eta }_{p}^{2}$ = 0.348. However, we found a significant interaction effect between set size and target presence, *F*(2, 62) = 14.558, *p* < 0.001, ${\eta }_{p}^{2}$ = 0.320. The simple main effects showed that response time increased with set size in target-absent trials, *F*(2, 62) = 20.921, *p* < 0.001, ${\eta }_{p}^{2}$ = 0.403, but not in target-present trials, *F*(2, 62) = 1.506, *p* = 0.230, ${\eta }_{p}^{2}$ = 0.046. One possibility is that the blur diminished the contrast between vertical and horizontal bars. In target-present trials, participants could find the target efficiently. In target-absent trials, however, they would rather scan items serially to ensure the correctness of their response. In the inefficient search task (Experiment 1c), the main effect of set size was significant, *F*(2, 58) = 65.282, *p* < 0.001, ${\eta }_{p}^{2}$ = 0.692. Experiment 1d tested the effect of distractor heterogeneity using the same stimuli as the 18-item arrays of Experiment 1c except that the distractors were rotated to different angles. When we compared the response time of Experiment 1d with that of Experiment 1c, we found that the response time was significantly longer in Experiment 1d, *F*(1, 29) = 93.609, *p* < 0.001, ${\eta }_{p}^{2}$ = 0.763, confirming the effect of distractor heterogeneity. Lastly, Experiment 1e tested for target priming effects on visual search performance. In this variant, the target could differ from trial to trial. Participants were asked to report the identity of the color singleton in the display. Even though the task was easy, participants were faster if the current target was the same color as in the preceding trial, *t*(31) = 4.270, *p* < 0.001, *d* = 0.755, 95% CI [0.074, 0.208]. In summary, the results replicate the main findings obtained on classic visual search tasks^8, 10, 12, 20–22^.

### Experiment 2: Search guidance by objects semantics

**Figure 4:**
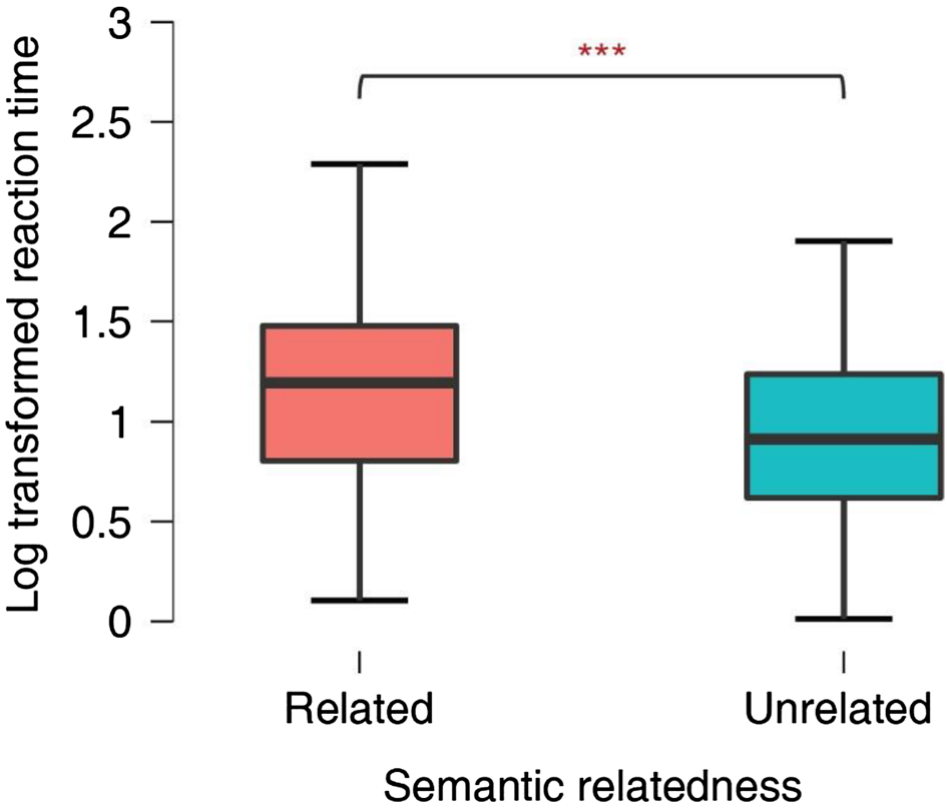
The box plot of log-transformed reaction time in semantic related and unrelated arrays. Participants spent more time to detect the target when the target was semantically related to the distractors, ***p < 0.001.

Experiment 2 attempted to replicate the results of Cimminella et al.^23^ which showed that participants respond faster to a target item embedded among semantically unrelated than related distractors. In this experiment, the printed name of a target object was briefly presented in the center of the screen, followed by an array of visual stimuli containing distractors drawn from the same or a different semantic domain. The image matching the target name was present in half of the trials and participants were instructed to report its presence or absence by pressing the corresponding key and, if reported present, indicate its location. The mean response accuracy was 0.91 (SD = 0.12) for semantically related trials and 0.90 (SD = 0.10) for semantically unrelated trials, which is similar to the results of the eye tracking study (M = 0.93, SD = 0.26). The effect of semantic relatedness was tested on the subset of correct trials using linear and generalized linear mixed-effects models. The fixed effect was semantic relatedness and the random variables, both as slopes and intercepts, were participant and item. We found the main effect of semantic relatedness on the log transformed reaction time, β = 0.22, SE = 0.035, *t*(27.388) = 6.314, *p* < 0.001. When the target was semantically related to distractor items in the display, it would take longer time to be found than when it was a semantic singleton (Fig. 4), indicating that visual attention can be guided by high-level attributes such as object semantics. We further analyzed search latency, defined as the time from the first touch on the screen to the first tracking point falling in the target area, and the probability of the first attended object, which is a binary variable indicating if the first object the participants touched was the target (1) or not (0). The search latency was much shorter in semantic unrelated arrays than in semantic related arrays (semantic unrelated: M = 894, SD = 765; semantic related: M = 1399, SD = 1092; linear mixed model: β = 455.84, SE = 84.10, *t*(27.64) = 5.42, *p* < 0.001). The effect of the probability of first attended object was not significant although the mean probability of the first touch directed to the target was higher in semantically unrelated trials (semantic unrelated: M = 0.369, SD = 0.483; semantic related: M = 0.319, SD = 0.467; generalized linear mixed model: β = − 0.3656, SE = 0.2, z = − 1.432, *p* = 0.152). These results are consistent with those of Cimminella et al.^23^ and indicate that standard overt visual search and visual search with the digit-tracking method detect similar effects of semantic relatedness on the allocation of visual attention.

### Experiment 3: Visual search in natural scenes

Experiment 3 tested visual search performance of participants looking for a pedestrian in an urban landscape, using stimuli developed by Ehinger et al.^24^. Since the database, including participants’ eye movement records, was made available online by the authors, we were able to make detailed comparisons between visual search results obtained with the digit-tracking method and standard eye-tracking.

### Exploration distance and response time

**Figure 5:**
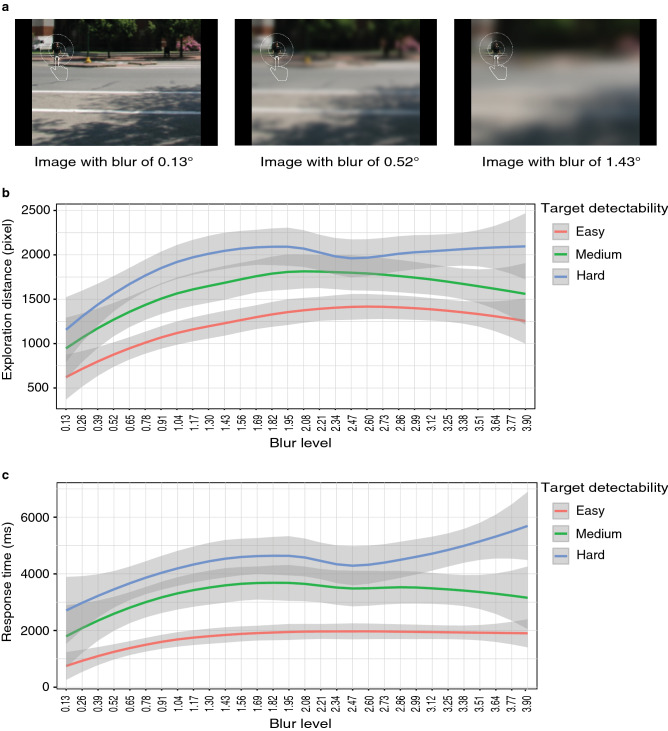
The stimuli and results of the Experiment 3a. (**a**) The same image superimposed by three different levels of blur. (**b**,**c**) Show how exploration distance and response time change along with the blur level.

Natural scenes generated more visual exploration than search arrays and allowed us to test the effects of several variables, notably the effects of target detectability and blur level (as defined by the standard deviation of the Gaussian blur filter). In Experiment 3a, we tested the impact of a large range of blur levels on search performance. Target detectability was determined and classified as low, medium or high based on response times measured on unblurred scenes by Ehinger et al^24^. The scene remained displayed until the participant's finger reached the target (a pedestrian) or had traveled a maximum allowed exploration distance Participants then ended the trial by pressing a key to signal whether or not they had detected the target. The small minority of trials (3.33%) in which participants reached the maximum distance of exploration and did not detect the target were excluded from statistical analyses. Dependent variables, i.e., exploration distance and response time were converted to logarithm to meet the assumption of normality. Participants explored longer distances if the target was more difficult to detect, *F*(2, 1074) = 12.543, *p* < 0.001, ${\eta }_{p}^{2}$ = 0.023, and higher blur levels also generated longer exploration distances, *F*(29, 1074) = 3.503, *p* < 0.001, ${\eta }_{p}^{2}$ = 0.086. Logically, response times were in keeping with exploration distances (Fig. 5c), with significant main effects of target detectability, *F*(2, 1074) = 37.987, *p* < 0.001, ${\eta }_{p}^{2}$ = 0.066, and blur level, *F*(29, 1074) = 2.506, *p* < 0.001, ${\eta }_{p}^{2}$ = 0.063. There was no significant interaction between target detectability and blur level on exploration distance, *F*(58, 1074) = 0.765, *p* = 0.902, ${\eta }_{p}^{2}$ = 0.040, and response time, *F*(58, 1074) = 0.994, *p* = 0.49, ${\eta }_{p}^{2}$ = 0.051, which means that the effects of blur level were similar in images of different target detectability.

Visual inspection of data showed that after a steady increase, exploration distances (and response times) gradually reach a plateau at high blur levels (Fig. 5b,c). So, further analyses focused on the trend analysis of blur level alone using orthogonal polynomial contrasts. The result shows linear effects and quadratic effects of blur level on both dependent variables (exploration distance: linear, *t* = 5.010, *p* < 0.001, quadratic, *t* = − 3.404, *p* < 0.001; response time: linear, *t* = 4.024, *p* < 0.001, quadratic, *t* = − 2.581, *p* = 0.01), in line with the observation. High blur will not only mask the location of target, but also reduce the information provided by the context. The flattening of the exploration distance and response time curves suggests that beyond a certain level of blur, the quantity of residual information is identical between two levels of blur.

Since we had access to the published eye-tracking data recorded in Ehinger et al.^24^, we also computed the eye-tracking reaction time and exploration distance for the subset of images used in the present study. The exploration distances for the easy, medium and hard target detectability were 664 (SD = 251), 967 (SD = 410) and 1248 (SD = 453) pixels. The reaction times for the three groups were 722 (SD = 215), 1031 (SD = 271) and 1281 (SD = 312) ms, respectively. Ocular exploration distance and reaction time were significantly correlated, *r*(1258) = 0.772, p < 0.001. In our task, the exploration distances for easy, medium and hard target detectability were 1218 (SD = 814), 1605 (SD = 1064) and 1924 (SD = 1100) pixels. The response times were 1749 (SD = 1561), 3275 (SD = 3345) and 4385 (SD = 3520) ms, respectively. Finger exploration distance and response time were significantly correlated, *r*(1252) = 0.694, p < 0.001. Slower digit-tracking responses were expected given the higher inertia of the finger compared to the eyes and exploration distance and response times are averaged over the entire range from very light to very heavy blur levels. Also, differences in task instructions and response mode should also be considered. Indeed, whereas in the eye tracking study participants were only asked to report target presence/absence by a keypress, the instruction given to participants in Experiment 3a required them to localize explicitly the target by pointing at it, which may be a slower process. Despite these small differences in procedure and the overall slower response times for digit-tracking, the relative patterns were similar to those obtained with eye tracking.

### Characterization of attention maps

**Figure 6:**
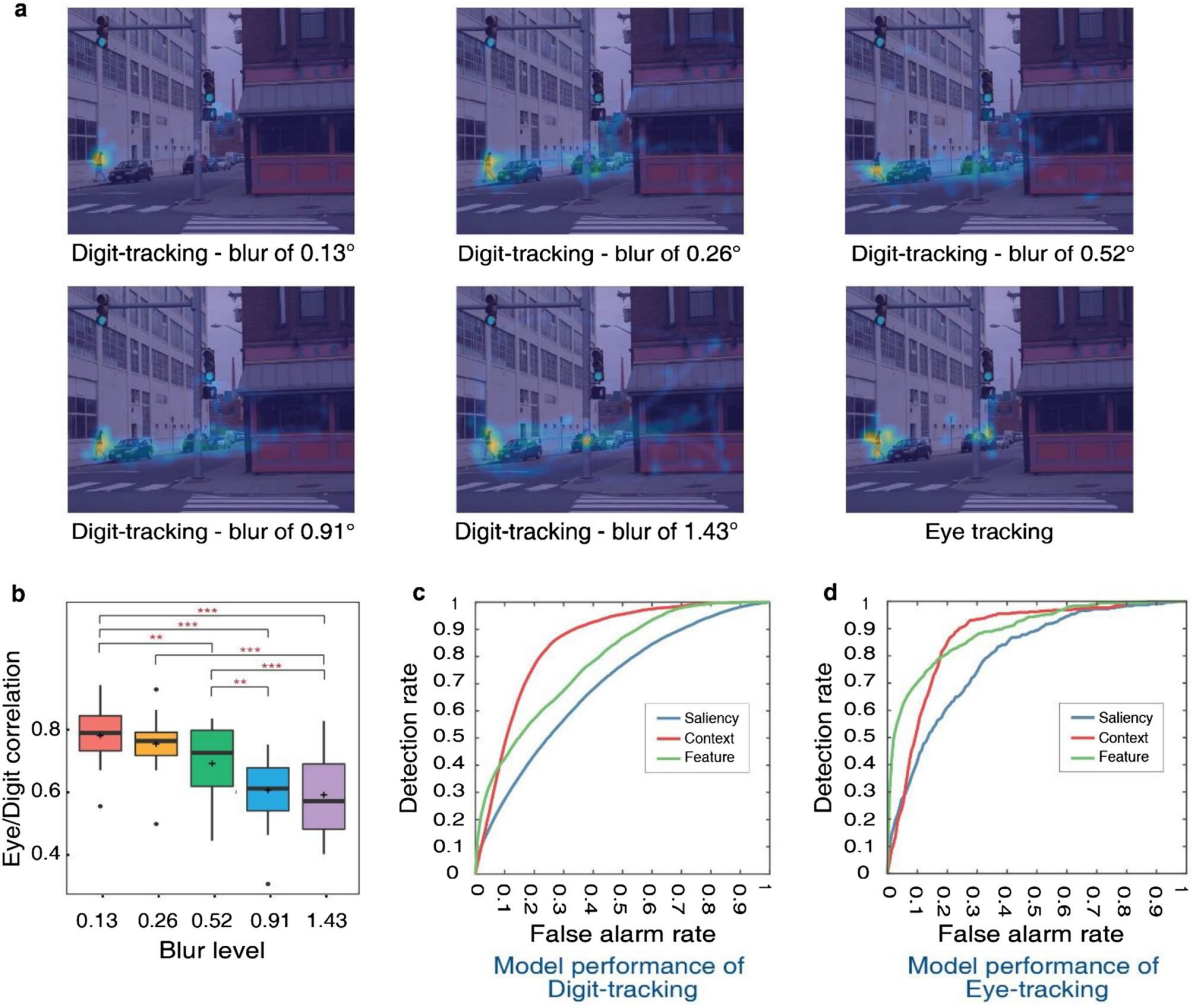
Results of the Experiment 3b. (**a**) Examples of digit-track attention maps at different blur levels and the eye tracking attention map computed from the Ehinger et al.^24^ (**b**) The boxplot of correlation coefficients between eye-tracking maps and digit-tracking maps, **p < 0.01, ***p < 0.001. (**c**,**d**) show the prediction performance of different models on digit-tracking data and eye tracking data.

Experiment 3b replicated the natural scene search task with a larger group of subjects and a subset of blur levels in order to generate a sufficient large number of exploration maps per scene and thus enable computing correlations between digit-tracking and eye-tracking explorations. We selected five blur levels and assigned these to 20 images across 75 participants in Latin square design, enabling us to get 15 data samples for each picture at each blur level. To compare exploration patterns obtained with the two methods, we generated attention maps from digit tracking data and compared them to those derived from corresponding eye tracking data available online^24^. Attention maps show a distinct hot spot at the target location in all conditions. However, participants also explored other candidate locations and could be attracted by objects looking like a human shape, with both testing methods (Fig. 6a). We computed the correlation between digit tracking maps at each blur level and eye tracking maps for the 20 images. Result of one-way ANOVA with Greenhouse–Geisser correction showed a significant main effect of blur level, *F*(2.862, 54.383) = 22.052, *p* < 0.001, ${\eta }_{p}^{2}$ = 0.537, indicating that two tracking methods show stronger correlations when blur is lighter (Fig. 6b). When pictures are masked by heavy blur, only the low spatial frequency content of the picture can be used to make the first inference about the pedestrian location, so participants have to use their prior knowledge about outdoor scenes to predict the potential location of target, causing more exploration traces on areas which are likely to contain pedestrian (e.g. near roads, cars, doors, etc.). The issue of search guidance is addressed next.

### Comparison with models of search guidance

We examined putative mechanisms underlying visual search by testing computational model predictions of the empirically observed exploration maps. Three sources of attentional guidance were considered: saliency, target features and scene context. Saliency represents a mixture of local image features like color and orientation. The target in this task is a pedestrian so we used a person detector algorithm to compute target features^25^. Computing scene context involves estimating the holistic spatial frequencies in the image. We tested the prediction performance of these single-source models on our digit-tracking data and compared it to predictions of the eye-tracking data of Ehinger et al.^24^. We plotted the average receiver operating characteristic (ROC) curves across five blur levels for each model (Fig. 6c shows the ROC curves of the first blur level, i.e., 0.13°). Detection rate, on the y-axis, corresponds to the proportion of tracking points falling within the prediction map generated by the models, while false alarm rate, on the x-axis, corresponds to the proportion of the image area selected by the map. Then we reported their area under the curve (AUC) as an index for the prediction accuracy and conducted the ANOVA on the AUC. The main effect of model was significant, *F*(2, 38) = 65.564, *p* < 0.001, ${\eta }_{p}^{2}$ = 0.775, so we performed post hoc tests using Bonferroni correction. The scene context model outperformed the target feature model, *t*(19) = 4.074, *p* < 0.001, 95% CI [0.021, 0.086], and the saliency model, *t*(19) = 11.305, *p* < 0.001, 95% CI [0.115, 0.181]. And the feature model had a better performance than the saliency model, *t*(19) = 7.231, *p* < 0.001, 95% CI [0.062, 0.128]. We performed the same analysis and obtained similar results on eye tracking data (Fig. 6d), *F*(2, 38) = 6.983, *p* = 0.003, ${\eta }_{p}^{2}$ = 0.269. The saliency model obtained a poorer performance than the context model, *t*(19) = − 2.746, *p* = 0.028, 95% CI [− 0.139, − 0.006], and the feature model, *t*(19) = − 3.568, *p* = 0.003, 95% CI [− 0.160, − 0.028]. However, we found no significant difference between the feature model and the context model, *t*(19) = 0.823, *p* = 1.000, 95% CI [− 0.044, 0.088].

As previously reported^24, 26–28^, pure saliency models fail to account for visual search patterns, in contrast to scene context and target features which do a relatively better job. We further tested models’ performance as a function of blur level. The ANOVA results revealed that prediction accuracy of all models except the scene context model decreases linearly and significantly as blur gets heavier [saliency: *F*(4, 76) = 16.696, *p* < 0.001, ${\eta }_{p}^{2}$ = 0.468; target feature: *F*(4, 76) = 18.091, *p* < 0.001, ${\eta }_{p}^{2}$ = 0.488; context: *F*(4, 76) = 2.130, *p* = 0.085, ${\eta }_{p}^{2}$ = 0.101]. Saliency and target feature involve computation of local features, which are most degraded by heavy blur. However, context which captures global scene characteristics is more robust to decreased image resolution. The fact that processing of such information remains useful under high image degradation likely explains why the context model is a strong predictor of scene exploration in unblurred images as well: rapid processing of global information with the resolution of the peripheral retina provides strong and efficient guidance toward regions that are most likely to contain a target.

### Human visual acuity and simulated peripheral vision

**Figure 7:**
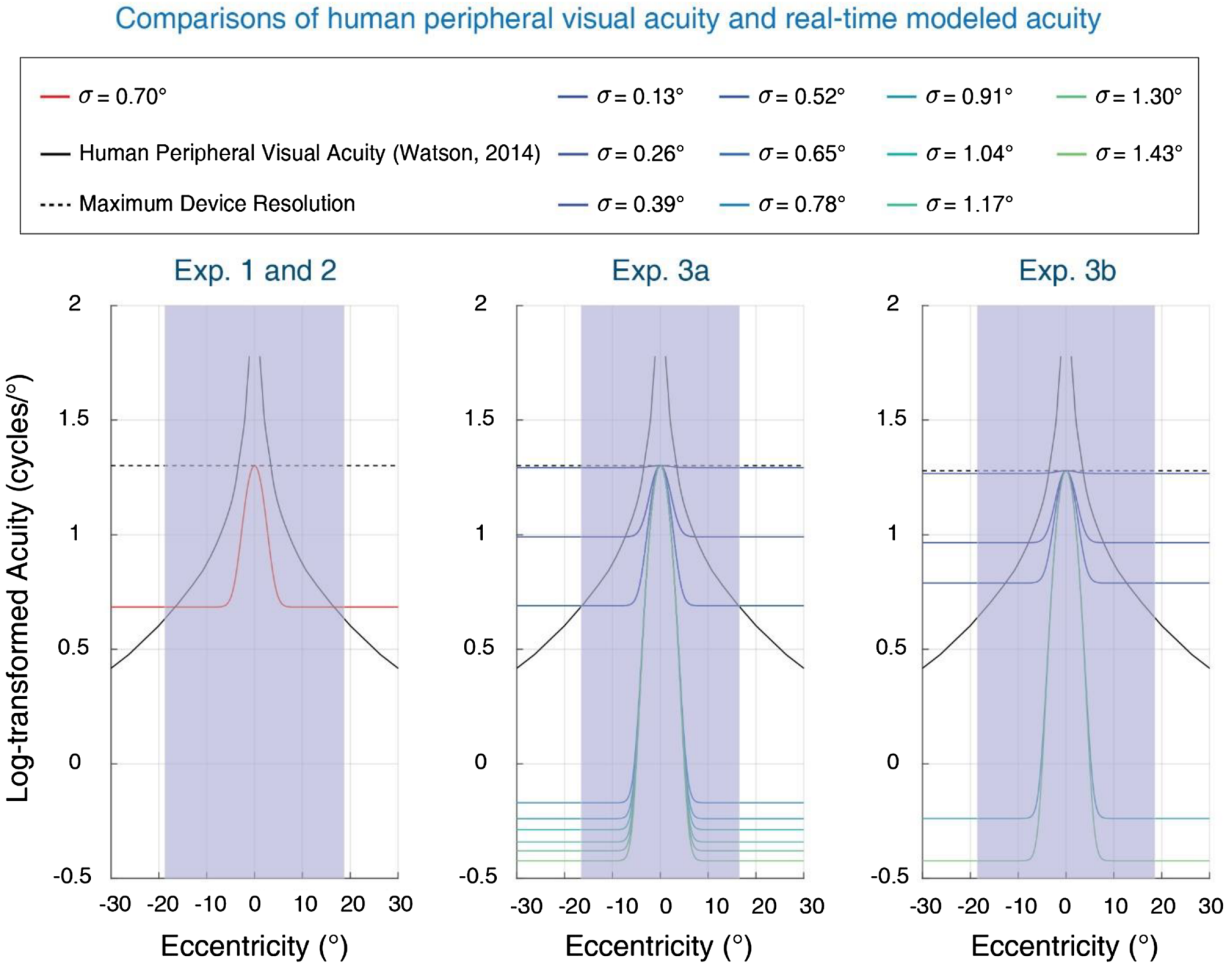
Comparisons of human peripheral visual acuity and the visual acuity simulated by digit-tracking in three experiments. Experiments 1 and 2 used the same blur level as Lio et al.^4^. Experiment 3a tested 30 blur levels but only the first 11 levels were shown here. Experiment 3b used 5 blur levels selected from Experiment 3a. Purple areas represent the screen eccentricity.

Since visual search performance is directly impacted by the blur parameters, we estimated the actual human peripheral visual acuity using the formula described in Watson^29^ (Fig. 7, continuous black line), and compared it to the estimates of maximal possible acuity in the digit-tracking task. The upper bound of simulated acuity is defined by the maximum resolution of the device used to present the stimuli (Fig. 7, dotted black line). We found that whatever the level of blur was used in current study, the quantity of information exploitable in peripheral vision in the digit-tracking tasks was systematically lower than the quantity of information would be available if the images were not affected, within a broad cone of visual eccentricities. Nevertheless, we still observed comparable digit-tracking results to eye-tracking results, which implies that the search behaviors can be modulated by very limited information.

## Discussion

The digit-tracking approach allows testing visual-attentional processing at the spatial resolution of the peripheral retina and thus reveals how low-resolution signals guide attention, and eventually trigger finger exploration when more detailed foveal analysis is needed. Our results show that these two aspects can be dissociated depending on the nature and discriminability of the searched items. With standard visual arrays composed of single features or combination of features, like abstract geometric shapes and highly contrasted colors, participants’ performances with the digit tracking method largely reproduced classical findings regarding the search array characteristics that determine efficient and inefficient search^8, 10, 20–22^. However, under our testing conditions, participants scarcely needed to (digitally) foveate stimuli suggesting that such tasks are largely performed at low resolution. The search for a color-defined target deserves special consideration. We approximated the low visual acuity of the peripheral retina by applying a Gaussian blur filter of constant width across the display. However, we did not attempt to mimic the color sensitivity function of the retina, a considerably bigger challenge. Indeed, since color contrast sensitivity decreases very gradually from fovea to periphery^30^, its implementation requires the application of an adaptive color filter of variable width that should be dynamically updated as the finger moves across the display. Future studies with our method might therefore consider avoiding color as a key target feature or favoring grayscale images to ensure that finger tracking is as faithful as possible to actual eye explorations. Factors like distractor heterogeneity, search history, and semantic relatedness were also shown to determine search reaction time and accuracy when participants were tested with the digit-tracking interface in the same manner as more traditional approaches. But again, only for the most difficult conditions, i.e. when search arrays contained pictures of real objects with small details, did participants need to resort to a finger-mediated exploration strategy. Using natural scenes as search stimuli, we observed similar results with the digit-tracking method as with classical eye-tracking^24^. Attention maps computed from the respective data sets were highly correlated and different sources of guidance made similar contribution with these two methods. Combining all the present evidence, we conclude that digit-tracking is a reliable tool that could be useful in studies of visual search with complex or naturalistic stimuli.

The gaussian blur used by the digit-tracking method aims to simulate the spatial resolution of the peripheral retina. The simulated visual acuity in the present study was actually below the “physiological” level. Increasing the blur level can further diminish the detectability of small details. Nevertheless, we found that, even at high blur levels, participants were able to extract useful scene information. Evaluation of different computational models of attention shows that blur increase undermined search guidance by saliency and target features, but guidance by scene context was unaffected, which highlights the important role played by contextual information in natural visual search. Research in visual cognition increasingly focuses on real-world scene analysis because of its relevance to people’s actual visual experience in daily life. Several studies have demonstrated that scene guidance plays a vital role and may make the largest contribution to natural visual search^5, 8, 17, 31^. Importantly, scene context can be extracted at a coarse spatial scale, which can explain why it is relatively insensitive to the blur level^32^. Observers can grasp the global structure of the stimuli before touching the screen, which can guide their attention to areas likely to contain a target. At this early processing stage, object features are not as important as in the case of abstract or artificial stimuli. Therefore, digit-tracking can provide a new perspective to resolve the debate on the contribution and relative priority of bottom-up and top-down factors in visual search. There are, of course, potential limitations to using this method. In particular, the eye-to-screen distance was not accurately controlled and we did not attempt to match the size of the foveal visual field of each participant. Although our previous results show that attention maps obtained with the current implementation of digit-tracking and with standard eye-tracking are highly correlated^4^, there could be uncontrolled measurement errors. Such noise sources could be reduced using a chin rest and customized foveal apertures for applications that require a highly accurate simulation of the visual acuity gradient.

In addition to its potential value for vision research, the digit-tracking method can also be beneficial in practical applications where large-scale data acquisition is needed. Lio et al.^4^ highlighted the usefulness of digit-tracking in three domains: cognitive, computer, and medical science. Medical applications are worth emphasizing here because visual search tasks are often used to test patients with psychiatric or neurological disorders. Performance on visual search has been proved to be early or auxiliary diagnostic markers of Alzheimer’s disease^33–37^, autism spectrum disorder^38, 39^, affective disorders^40^, schizophrenia^41^ and visual neglect^42^. For example, Gonçalves et al.^33^ reviewed literature concerning the changes of visuomotor patterns in mild cognitive impairment and Alzheimer’s disease, and concluded that eye movement analysis can help detect early signs in the course of this disease. Gliga et al.^38^ demonstrated that visual search performance in 9-month-old infants can predict a higher level of autism symptoms at 15 months and 2 years. Due to its simplicity, portability and calibration-free implementation, digit-tracking has an advantage over traditional eye-tracking methods in patient testing, especially for children and the elderly. Unlike neurotypical people, patients possibly have difficulties completing eye movement tasks or tolerating the measurement device. Medical personnel may not have sufficient knowledge or resources to operate eye-tracking devices or process the resulting data. Therefore, using digit-tracking can bring much needed convenience in clinical contexts. Further work should be undertaken to build and validate standard visual search tests using digit-tracking.

To sum up, we evaluated the digit-tracking approach using a range of visual search tasks. The results presented in the current study are comparable to those reported in previous studies using more standard approaches. Since the visual search paradigm is still widely used in basic and applied vision science, digit-tracking could represent a valuable new tool for future research in this field.

## Methods

### Participants

A group of thirty-two participants (mean age = 19.2, SD = 1.4) were recruited for Experiments 1a, 1b and 1e; thirty-one of them participated in Experiment 2 as well. Another group of thirty participants (mean age = 19.4, SD = 1.7) were recruited for Experiments 1c and 1d. Fifteen (mean age = 25.3, SD = 2.7) and seventy-five (mean age = 22.5, SD = 2.5) participants were recruited for Experiments 3a and 3b respectively. In Experiment 3, to avoid having participants see the same image multiple times, we assigned blur levels to different images in a Latin square design, and we fixed the number of participants based on the number of blur levels used in this design. A larger number of participants was used in Experiment 3b in order to compute reliable average attention maps.

All but fifteen of the participants in Experiment 3a were recruited from South China Normal University. The fifteen other participants were recruited at the Institute of Cognitive Science Marc Jeannerod (CNRS) as part of a collaboration project conducted between the two institutions during which the first author was an exchange student. All participants gave written informed consent prior to participating, reported normal or corrected-to-normal vision, and reported no history of neurological or psychiatric disorders. All the experiments followed all guidelines and recommendations and were approved by the Ethics Committee of South China Normal University in accordance with the Declaration of Helsinki.

### Digit-tracking method

The digit-tracking method was implemented following the same framework described in Lio et al.^4^. Stimulus presentation, real-time picture modification and data recording were written using the Psychophysics Toolbox^43, 44^ for MATLAB (r2015a-the MathWorks, Inc.). The participant was instructed to sit in front of a tablet computer without any constraint. The median eyes-screen distance was 45 cm, in the range of 35 to 50 cm. In each trial, a green virtual button was displayed on a gray background at the screen center and the participant triggered the appearance of the blurred stimulus by touching this button. The picture degradation simulated low acuity of peripheral vision. By touching the screen, the participant could see a clear aperture area above the contact point, which simulated high acuity of foveal vision. The aperture was shifted upwards to prevent the participant’s view masking by the finger. Searching for the target could be accomplished by sliding the finger over the display to update the high-resolution aperture. Coordinates of the aperture’s center, regarded as the proxy for eye positions, were continuously recorded. The close coupling between finger and eye during digit-tracking visual search can be observed when the movements of both effectors are tracked. As an illustration, we used a scene camera to capture a participant’s finger movements during mock trials while her point of gaze was simultaneously recorded using a head-mounted infrared eye tracker^45^ (Pupil Labs, Berlin). The superimposed eye and finger trajectories are shown in Supplementary Videos 1–3.

In the implementation of digit-tracking method, three parameters have to be taken into consideration: the level of picture degradation simulating peripheral vision, the size of the simulated foveal area and the location of the foveal aperture with respect to the contact point. We used a Gaussian filter of size σ = 0.7° to mimic peripheral vision except in Experiments 3a and 3b in which we manipulated the blur level to test its effect on the search pattern. The simulated foveal vision was created by a Gaussian aperture window of size σ = 1.9° and the distance between contact point and the center of foveal vision was 1.4°.

### Task design and procedure

Stimuli in Experiment 1 were arrays of abstract items like colored squares, oriented bars and letters on a grey background (Fig. 2a). Arrays were presented within an area of 21.4° × 21.4° of visual angle at the center of the screen. The size of items varied in different arrays (colored squares: 1.11°*1.11°, oriented bars: 1.67°*0.56°, letters: 2.23°*1.67°). Target locations were balanced across trials to avoid any directional bias. In Experiments 1a, 1b and 1c, set size was used as a within-subject variable to examine search efficiency for the different classes of stimulus arrays. There were three set sizes (6, 12 and 18) and 10 trials per set size for a total of 30 trials per task. Experiment 1a was a color feature search task in which participants had to determine if a red square was present among blue squares. Experiment 1b was an orientation feature search task in which participants had to determine if a horizontal bar was present among vertical bars. Experiment 1c was an inefficient search task in which participants had to report if a letter T was present among Ls of different colors. The stimuli and task in Experiment 1d were similar to those in Experiment 1c, except that the distractors were rotated by different angles. Ten trials of 18-item arrays were performed and search performance was compared to the corresponding trials in Experiment 1c in order to test the effect of distractor heterogeneity. In Experiment 1e, participants saw 18-item arrays of colored letters and needed to report the identity of the color singleton in the display. The task comprised 21 trials, ten of which contained the same target color as on the previous trial to test for priming effect on search time. In all these tasks, the trial ended when the participant pressed the appropriate key on the keyboard to report either target presence/absence or target identity (T or L).

Stimuli in Experiment 2 were arrays of real-world objects and animals developed by Cimminella et al.^23^ using images selected from the Bank of Standardized Stimuli (BOSS) database^46^. We used a subset of 96 7-item arrays (64 target-present arrays and 32 target-absent arrays). In half of the target-present arrays, targets and distractors were semantically related and, in another half, they were semantically unrelated. Since all participants in this experiment were Chinese, we validated the manipulation of Chinese semantic relatedness using sematch library in Python. Applying a knowledge-based approach^47^, we confirmed that semantic similarity between targets and distractors was significantly higher in semantically related than unrelated arrays, *t*(191) = 10.422, *p* < 0.001, *d* = 0,75, 95% CI [0.175, inf]. Figure 2b shows examples of a semantically related array and a semantically unrelated array. Objects could be used repeatedly, so we separated target-present arrays into two groups of 32 arrays which didn’t share any object. Each group contained 16 semantically related and 16 semantically unrelated arrays. Each participant saw either group of target present arrays and 32 target-absent arrays to make sure each object only be shown to each participant once. Arrays were presented on a white background within an area of 28.07° × 21.40° of visual angle at the center of the screen. Objects had a size of 4.18° and were arranged on an imaginary circle which had a fixed radius of 9.62°. Target locations were balanced across trials to avoid any directional bias. Each trial began with the presentation of a cue word for 800 ms, followed by a fixation point for 100 ms and then the search array. The participants were instructed to determine whether the target was present or not via keypress within 10 s. If the response was present, a number array would be prompted to ask the participants to confirm the location of target.

Stimuli in Experiments 3a and 3b were selected from a picture database used in Ehinger et al.^24^. This database was published online, including eye-tracking data recorded during a visual search task using these pictures. We selected a subset of the pictures which had to meet two criteria: small standard deviation of reaction time and accuracy over 90 percent, assuring that observers’ performance on these images was homogeneous. Experiment 3a included two within-subject variables: blur level and target detectability. Blur level ranged from σ = 0.13° to 3.9° in steps of 0.13° (sigma value of the Gaussian blur filter), yielding thirty levels. Ninety pictures were classified into easy, medium and hard levels of target detectability based on the average response times reported in the published database. Since each image was to be presented only once to each participant, blur levels were assigned to different images across the 15 subjects in a Latin square way. During the presentation, images were resized to fit one side of the screen with the ratio of height to width fixed. Participants were instructed to find a pedestrian in the natural scene by exploring the image, pointing at the target when they found it, and then confirming whether or not they had seen it by pressing the corresponding response key. The image presentation ended when the finger remained on the target for 700 ms or had reached the maximum allowed distance of exploration (70° of visual angle), after which participants were allowed to give the detection confirmation response. The finger dwell time requirement served to prevent premature termination of the trial (e.g., the finger sliding past the undetected target). Response time for detected targets was computed as the time elapsed between the stimulus onset and the time at which the finger settled on the target. Exploration distance was computed as the cumulative travel distance between the first finger contact on the display surface and the time at which the finger settled on the target.

In Experiment 3b, 20 pictures were selected respecting the two criteria mentioned above. We chose five relevant blur levels (i.e. 0.13/0.26/0.52/0.91/1.43 degree) based on the results of Experiment 3a and assigned them to different pictures across subjects in a Latin square way, assuring that we got 15 data samples for each picture at each level. We used the same procedure as in Experiment 3a except that there was no dwell time requirement. Participants were instructed to explore the image and a trial ended when the participant pressed the keyboard or the exploration distance reached 70° of visual angle.

This modification to the procedure between Experiments 3a and 3b was motivated by the unexpected presence of invalidly terminated trials in Experiment 3a. Indeed, some participants reported that the trial did not end even though they had found the target, most likely because they stopped exploring while the finger was still slightly away from the target. When this happened, they adopted the implicit strategy of terminating stimulus presentation by reaching the maximum travel distance with random movements and then pressing the “target detected” response key. Since one of the main objectives of Experiment 3b was to analyze attention map profiles, we wished to collect digit-tracking data uncontaminated by such non-exploratory movements.

### Data analyses

In Experiment 1, we used parametric tests to analyze the reaction time. Experiment 2 was adapted from Cimminella et al.^23^. For comparison purpose, we used the same analysis procedure described in their work, which is using linear and generalized linear mixed-effects models to analyze the log-transformed reaction time, search latency and probability of the first attended object being the target. In Experiment 3a, we analyzed reaction times and exploration distances using parametric tests and orthogonal polynomial contrasts. Trials considered as invalid (3.37% of all recorded trials) were excluded from the analyses. These were improperly terminated trials that yielded unreliable estimates of exploration distance or reaction time, as described in the previous section. In Experiment 3b, we computed the empirically observed exploration maps (i.e., attention maps), so that we can compare the exploration from digit-tracking and eye tracking. Also, we built computational models to predict the participants’ attention and compared the performance of different models.

### Linear and generalized linear mixed-effects model

This procedure was implemented by the lmerTest package^48^ in R (version 4.0.3). We used linear mixed models (LMM) to analyze the continuous variables (i.e., log-transformed reaction time and search latency) and generalized mixed model (GLMM) to analyze the binary variable (i.e., the probability of the first attended object). The fixed effect was semantic relatedness. The random effects were, both as intercepts and slopes, were Participant and Target. The models were fitted using restricted maximum likelihood approach and p-values were computed using Satterthwaite's method (LMM) and asymptotic Wald tests (GLMM).

### Attention maps

Attention heat maps for Experiment 3 data were computed as described in Lio et al.^4^. Briefly, coordinates of tracking points were transformed from the screen space to the picture space. Attention map for each subject and each picture, i.e. the probability density of exploration, was calculated from the tracking data using kernel density estimation with a Gaussian kernel weighted by duration and a bandwidth of 1° of visual angle. Each subject-level density was then normalized using the Min–Max normalization. Finally, group-level maps were generated by averaging scaled subject-level densities and normalized using the same normalization procedure. Attention maps for eye-tracking data from the Ehinger et al.^24^ database were computed from segmented eye fixations following the same procedure. Pearson correlations between eye tracking maps and digit tracking maps at each blur level were computed and statistical hypothesis tests on correlation coefficients across different blur levels were conducted.

### Computational models of attention

To investigate what kind of information could guide observers’ attention, we used the framework originated from Torralba et al.^17^ and described in Ehinger et al.^24^. To predict observers’ attention, these authors compared models built on three sources of guidance: saliency, target features and scene context. Map matrix of each single-source model were available online. Saliency was computed by estimating the distribution of local features (e.g. color, orientation and scale). Locations differ in local features from neighboring areas were more likely to be salient. Torralba et al. fitted a multivariate power-exponential distribution to compute the probability of predicting tracking points at each pixel. Target features were computed using a classified-based algorithm called person detector^25^. This detector adopted a scanning window method to extract features of each picture, then used a linear Support Vector Machine to classify each extract as target or background. The detector was tested by Ehinger et al.^24^ using different window sizes and here we only used the best performance model with a scanning window of 32*64 pixels. It assigned severeal detection scores to each pixel, and the target feature map was constructed by choosing the highest detection scores at each pixel. The saliency map and the target feature map were multiplied by a pretrained exponent and blurred by a Gaussian filter. Computing context required estimating global features which provided a holistic description of spatial frequencies and orientations of the picture. The model was trained to learn the association between global features and the location of target and to predict the presence of the target at each pixel in a new picture. To evaluate model performance, we used map matrix of different models to predict participants’ tracking points and generated the Receiver Operating Characteristic (ROC) curves for each image. The area under curves (AUC) were used to evaluate the prediction performance.

## Supplementary Information

Supplementary Information.

## Data Availability

The datasets generated and analyzed during the current study are available from the corresponding author on reasonable request.
